# Bioinspired Processing of Keratin into Upcycled Fibers through pH-Induced Coacervation

**DOI:** 10.1021/acssuschemeng.2c06865

**Published:** 2023-01-25

**Authors:** Jianwu Sun, Guillermo Monreal Santiago, Feng Yan, Wen Zhou, Petra Rudolf, Giuseppe Portale, Marleen Kamperman

**Affiliations:** † Polymer Science, Zernike Institute for Advanced Materials, University of Groningen, Nijenborgh 4, 9747 AG Groningen, The Netherlands; ‡ Surfaces and Thin Films, Zernike Institute for Advanced Materials, University of Groningen, Nijenborgh 4, 9747 AG Groningen, The Netherlands; § Products and Processes for Biotechnology, Engineering and Technology Institute Groningen, University of Groningen, Nijenborgh 4, 9747 AG Groningen, The Netherlands; ∥ Macromolecular Chemistry and New Polymeric Materials, Zernike Institute for Advanced Materials, University of Groningen, Nijenborgh 4, 9747 AG Groningen, The Netherlands

**Keywords:** keratin, upcycling, coacervate, fibers, processing, regenerated, wet spinning

## Abstract

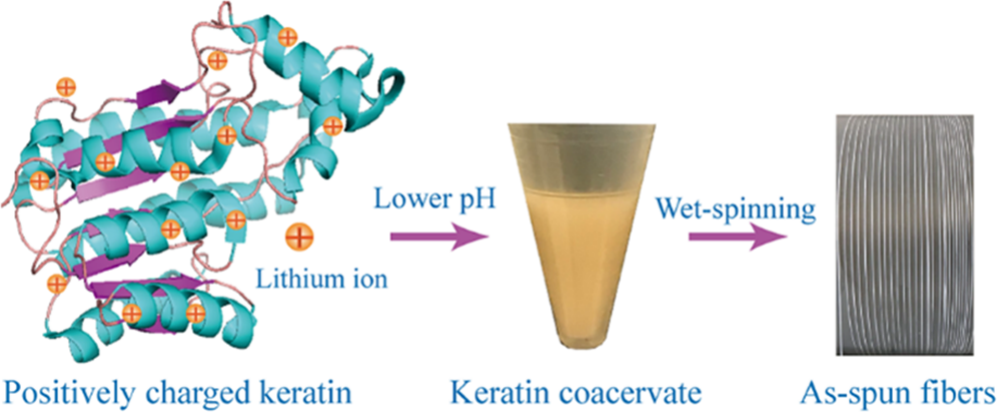

Keratin is an important byproduct of the animal industry, but almost all of it ends up in landfills due to a lack of efficient recycling methods. To make better use of keratin-based natural resources, the current extraction and processing strategies need to be improved or replaced by more sustainable and cost-effective processes. Here, we developed a simple and environmentally benign method to process extracted keratin, using HCl to induce the formation of a coacervate, a separate aqueous phase with a very high protein concentration. Remarkably, this pH-induced coacervation did not result in the denaturation of keratin, and we could even observe an increase in the amount of ordered secondary structures. The low-pH coacervates could be extruded and wet-spun into high-performance keratin fibers, without requiring heating or any organic solvents. The secondary structure of keratin was largely conserved in these regenerated fibers, which exhibited excellent mechanical performance. The process developed in this study represents a simple and environmentally friendly strategy to upcycle waste keratin into high-performance materials.

## Abstract 2

We report a simple and green method to process keratin into high-performance fibers through coacervate formation induced by a decrease in pH.

## Introduction

Growing concerns about the shortage and nonsustainability of resources have stimulated research to replace petrochemical plastics with bio-based materials. Proteins from natural resources have attracted much attention as green materials for various applications, including coatings, packaging, adhesives, cosmetics, and fibers, due to their excellent biocompatibility and good mechanical properties.^1−6^ Keratin, abundantly available through extraction from wool, feathers, and hair, is a particularly promising candidate to substitute oil-derived synthetic products.^7−9^ However, most keratin waste from the textile and poultry industry currently ends up in landfills and is not recycled. To change this and to make full use of keratin-based natural resources, new extraction and processing strategies are needed.

Keratin extraction from wool and feathers has been extensively studied in the past.^10−15^ During the extraction process, inter- and intramolecular interactions (mostly hydrogen bonds and disulfide bonds) need to be broken to facilitate the dissolution of keratin.^7−9^ More specifically, disulfide bonds can be reduced to thiol groups by reducing agents such as 2-mercaptoethanol, dithiothreitol (DTT), and sodium bisulfite, while hydrogen bonds can be disrupted by lithium bromide (LiBr), sodium dodecyl sulfate (SDS), and ionic liquids. However, the pure keratin obtained upon removal of these agents (e.g., by dialysis) is highly hydrophobic and has a low solubility both in water and most organic solvents. This poor solubility makes the fabrication of keratin and keratin-composite materials challenging, which is currently limiting the scope of keratin-based applications.^7^ To remedy the poor processability of keratin, a common strategy is to keep the compounds used in the extraction process in solution during processing.^7^ However, this normally leads to a loss of the original hierarchical structure of keratin, which impacts the final material properties.

In contrast to SDS and ionic liquids, using LiBr during extraction allows for the protofibril structure of keratin to be conserved, showing a higher potential to form materials with a restored hierarchical structure. LiBr solubilizes keratin through the adsorption of Li ions on the protein surface, which leads to positive charges.^16,17^ These positive charges result in repulsive forces between the keratin polypeptides, thereby enhancing their solubility. The keratin extracted in this way also has the potential of being processed in mild conditions through electrostatic complexation. Cera et al. used phosphate ions for this purpose, leading to shape-memory materials with a conserved hierarchical structure.^18^ However, their method only used the peptides of higher molecular weight, losing around one-third of the extracted keratin. As an alternative, we have recently reported a method where all of the extracted keratin could be processed through complexation with a synthetic polyanion, but the resulting materials had completely lost their hierarchical structure, in detriment of their mechanical properties.^19^ Furthermore, the fibers produced by these methods rely on the presence of LiBr in the final fibers, which would result in an additional environmental impact if produced on a large scale. To the best of our knowledge, there are currently no processing methods that are green, simple, and use all of the extracted keratin while conserving its hierarchical structure in the final materials.

Meanwhile, the technological challenges involved in the green processing of biopolymers, and in particular proteins, have already been overcome by many natural organisms. Spiders, caddisfly larvae, and velvet worms are able to produce strong and tough fibers. Recently, it has been suggested that these organisms use coacervate phases as intermediates toward the final material.^20,21^ Coacervation is a liquid–liquid phase separation into two immiscible liquid phases, often driven by electrostatic and/or hydrophobic interactions, which results in a dense phase (the coacervate) and a dilute phase.

In these natural systems, (gradual) changes in external factors, such as pH and ion concentration, seem to both drive the formation of coacervates and play a key role during their processing and solidification.^21−23^ For example, caddisfly larvae secrete fluid material that quickly solidifies when in contact with seawater with a higher pH. Similarly, the adhesive and mechanical performance of fibers from spiders, silk, and velvet worms are greatly affected by environmental changes.^20−23^ Factors such as the presence of salts, pH, temperature, ionic strength, and shear stress can greatly influence the spinnability of the dope and the mechanical properties of regenerated protein fibers.^24−31^

**Figure 1:**
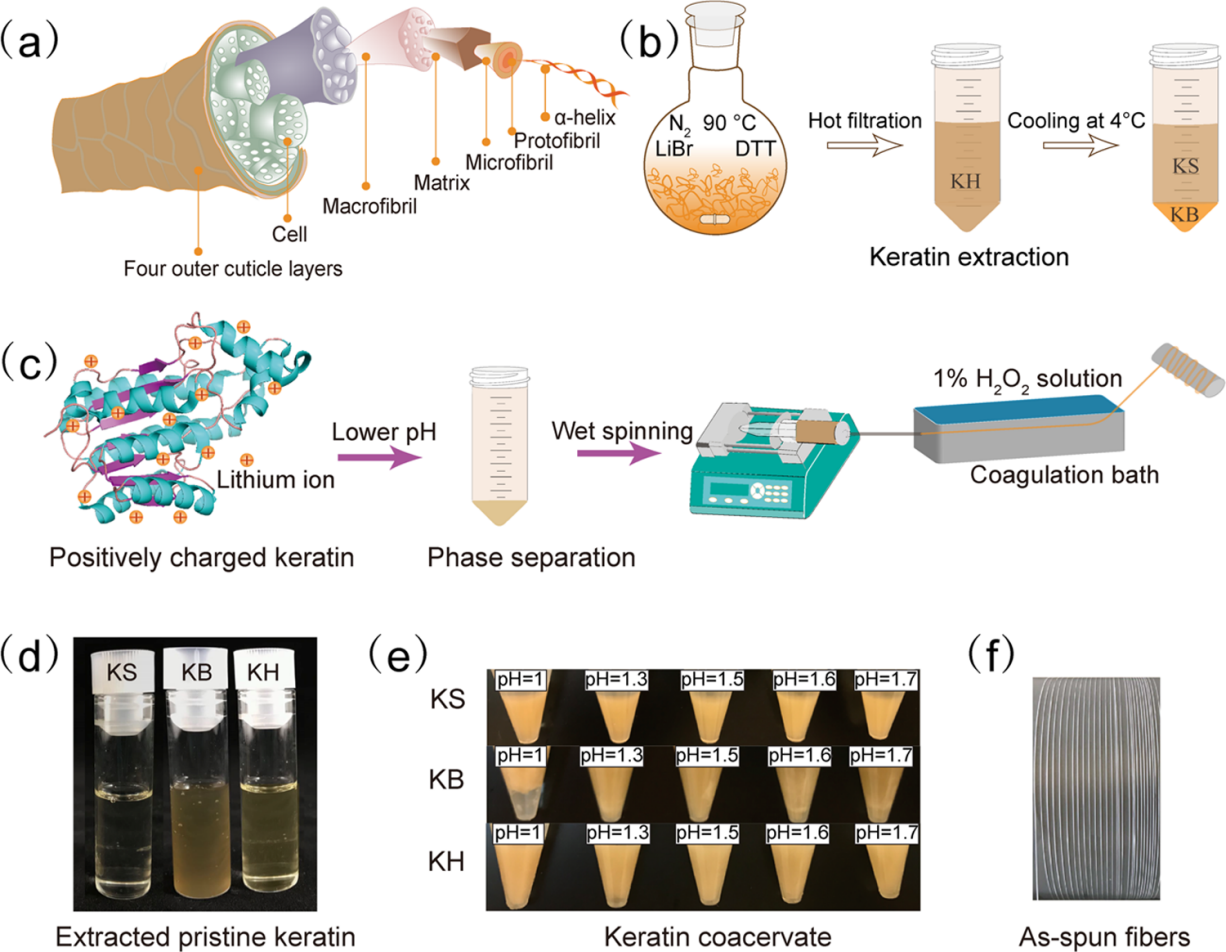
(a) Schematic diagram depicting the hierarchical structure of wool fibers; (b) extraction protocol of keratin from wool: keratin supernatant (KS), keratin bottom (KB), and keratin homogeneous (KH) phases; (c) schematic representation of keratin coacervate formation followed by wet-spinning processing; (d) photographs of pristine extracted keratins; (e) coacervates of KS, KB, and KH at different pH values; and (f) as-spun fibers.

Inspired by the formation of natural coacervates, here, we describe a simple and green method to process keratin, based on coacervate formation induced by a decrease in pH, followed by wet-spinning of the coacervate phase (Figure 1). Unlike traditional keratin processing methods, this protocol yields high-performance fibers without the use of organic solvents. First, we studied the formation of keratin-based coacervates under various conditions, their physiochemical properties, and the secondary structure of the keratin present in them. Second, we optimized the viscoelastic properties of these keratin coacervates by tuning the pH, to enable wet-spinning to produce regenerated fibers. Finally, we investigated the relationship between structural development and the mechanical performance of fibers, with an emphasis on the influence of drawing and orientation. We believe that the keratin coacervates developed in this study represent an unexplored strategy toward the manufacturing of biomimetic materials, and additionally, serve as a model to understand the phenomenon of coacervation in natural biomaterials.

## Experimental Section

### Materials

Lithium bromide (LiBr), dithiothreitol (DTT), hydrochloric acid solution (37–38%, HCl), and hydrogen peroxide solution (30%, H_2_O_2_) were purchased from Sigma-Aldrich and used without any further purification. Composite wool, consisting of 50% sheep wool and 50% alpaca wool, was purchased from a local shop.

### Keratin Extraction Protocol

Keratin extraction was performed as previously reported.^18^ Briefly, the wool was cut into small pieces and soaked in ethanol solution for 2 days. The wool was washed with water and allowed to dry in air at room temperature. Dried fibers (10 g) were immersed in a 150 mL aqueous solution of LiBr (8 M) and DTT (0.1 M) in a 250 mL flask. Subsequently, the keratin extraction was conducted at 90 °C for 36 h under N_2_ atmosphere. Finally, the keratin solution was obtained after fast hot filtration to remove insoluble residues. The homogeneous solution after filtration at room temperature is referred to as “keratin homogeneous” (KH). Upon cooling of the solution to 4 °C, phase separation was observed. The dilute upper phase was referred to as keratin supernatant (KS) and the dense, bottom phase was named KB.

### Preparation of Keratin Coacervate Induced by pH

All keratin solutions (KS, KB, KH, 10 mL) were acidified with a solution containing HCl and LiBr (pH = 0, [LiBr] = 8 M). By adding different amounts of this acidic solution to the keratin stocks, we reduced their pH while maintaining the concentration of LiBr (8 M) constant. The volumes of HCl solution were 1, 0.5, 0.3, 0.25, and 0.2 mL, and the corresponding resulting pHs were calculated to be 1.0, 1.3, 1.5, 1.6, and 1.7. Phase separation into a turbid dense phase (coacervate) and a clear dilute phase (supernatant) was observed for all solutions. The resulting coacervate dispersions were centrifuged at 750 g for 10 min to collect all of the coacervate phase at the bottom of the tube.

### Wet-Spinning of Keratin Coacervates

After centrifugation, the keratin coacervates were separated from the supernatant by decanting and directly used as spinning dope to produce regenerated keratin fibers. The wet-spinning device was custom-made and consisted of a syringe pump (AL-4000, WPI), a coagulation bath (1% H_2_O_2_), and a winding device. The diameter of the spinneret was 0.5 mm, and an extrusion speed of 0.01 mL min^–1^ was used. The collection rate of the roller was 13.4 mm s^–1^. The obtained as-spun fibers were manually stretched by 50% inside of the deionized water bath using tweezers and kept under tension in air until dry. As-spun fibers are referred to as unstrained fibers, which have a diameter of around 95 μm, and drawn fibers are referred to as strained fibers, which have a diameter of 80 μm.

### Characterization Techniques

#### Oscillatory Rheology

A rheometer (AR 4000, TA instruments) with a 20 mm parallel aluminum plate and a fixed gap of 500 μm was used to investigate the rheological behavior of the coacervates. The frequency sweeps were executed in the linear viscoelastic region (strain ≤ 5%) to determine the elastic modulus (*G*′) and loss modulus (*G*″) in a frequency range between 0.1 and 100 rad s^–1^. Time sweeps were performed to monitor the evolution of the moduli after a salt-switch at a fixed frequency of 1 Hz.

#### Thermogravimetric Analysis and Differential Scanning Calorimetry

The samples were dialyzed to remove LiBr and then freeze-dried before all DSC and TGA experiments. Thermogravimetric analysis (TGA) was performed on a TA Instruments TGA Q50. Samples were first heated to 100 °C and kept at 100 °C for 30 min to remove water. Next, the temperature was increased to 700 °C in N_2_ at a rate of 10 °C min^–1^. Differential scanning calorimetry (DSC) was carried out on a TA Instruments DSC Q100. Samples were heated at 2 °C min^–1^ from −30 to 300 °C with a modulation amplitude of 0.32 °C min^–1^ after being equilibrated at 100 °C for 30 min.

#### Attenuated Total Reflectance–Fourier Transform Infrared

Attenuated total reflectance–Fourier transform infrared (ATR-FTIR) spectra (resolution of 4 cm^–1^, 32 scans) were acquired using a Bruker Vertex 70 spectrometer in the wavenumber range of 400–4500 cm^–1^. Deconvolution based on curve fitting and fitting of the second derivative using Origin software was employed to analyze the amide(I) band (1600–1700 cm^–1^). The three main primary peaks were assigned to the secondary structures of keratin: β-sheet (1620 cm^–1^), α-helix (1650 cm^–1^), β-turn and random coil (1683 cm^–1^). The percentages obtained for each analyzed secondary structure correspond to their relative contributions to the amide(I) band. We take these percentages as a qualitative indication of the relative changes in secondary structures between different keratin samples.

#### Scanning Electron Microscopy

Scanning electron microscopy (SEM) images were collected using an FEI NovaNanoSEM 650 microscope. The acceleration voltage was 10 kV; a 20 nm thick gold film of was sputter-deposited on the sample surface to avoid charging effects. All keratin samples were dialyzed and freeze-dried before SEM observation.

#### X-ray Photoelectron Spectroscopy

X-ray photoelectron spectroscopy (XPS) was performed with an SSX-100 spectrometer (Surface Science Instruments), equipped with a monochromatic Al Kα X-ray source (*h*ν = 1486.6 eV). The measurement chamber pressure was maintained at 1 × 10^–9^ mbar during data acquisition. The photoelectron take-off angle was 37° with respect to the surface normal, and the diameter of the analyzed area was 1000 μm; the energy resolution was 1.26 eV (or 1.67 eV for a survey scan). All keratin samples were dialyzed against deionized water, lyophilized, and compressed to pills with the same conditions (RHC, 30 ton pillar press); a gold grid was placed on the top of the sample to avoid charging effects during XPS measurements. Spectral analysis included a Shirley background subtraction and fitting with peak profiles taken as a convolution of Gaussian and Lorentzian functions, with the help of the least squares curve-fitting program WinSpec (LISE, University of Namur, Belgium). Binding energies (BEs) were referenced to C 1s photoemission peak centered at 284.8 eV and are accurate to ± 0.1 eV when deduced from the fitting procedure. All measurements were carried out on freshly prepared samples, and three different spots were measured on each sample to check for homogeneity.

#### Tensile Testing

Tensile test curves were collected using a universal testing machine (Instron, 3400 series) equipped with a 10 N load cell. The experiment was carried out at a gauge length of 15 mm with a tensile speed of 10 mm min^–1^.

#### Polarized Optical Microscope

A polarized optical microscope (Nikon, Eclipse 600, POM) equipped with a Nikon camera (COOLPIX 4500, MDC Lens, Japan) was employed to characterize the birefringent properties of dried keratin fibers. A λ-tint plate (530 nm) was inserted in the POM to create contrast interference colors for all POM images. All fibers were oriented at 45° between crossed polarizers.

#### Wide-Angle X-ray Scattering

Wide-angle X-ray scattering (WAXS) measurements were performed at the Multipurpose Instrument for Nanostructure Analysis (MINA) beamline at the University of Groningen. The diffractometer is equipped with a Cu rotating anode (λ = 1.5413 Å); a sample-to-detector distance of 70 mm was used for WAXS. For the experiments, the fibers were glued on a stainless steel frame, which was then positioned parallel to the equator axis of the detector and perpendicular to the beam. The WAXS patterns were acquired using a Vantec500 Bruker detector. Calibration of the probed scattering angle range was achieved using the known diffraction pattern from an Al_2_O_3_ standard powder. Data analysis was performed using the Fit2D software. After radial integration around the beam center, the WAXS data are plotted against the modulus of the scattering vector *q* (in nm^–1^) = 4π sin(θ)/λ, where θ is half of the scattering angle.

## Results and Discussion

### Keratin Extraction

The first step in the upcycling of waste keratin is its extraction from the natural source. For this step, we utilized a previously published protocol based on the addition of DTT and LiBr.^18^ This protocol is known to solubilize keratin due to two processes: the reduction of the disulfide crosslinks by DTT and the adsorption of Li^+^ ions on the surface of the keratin fibers, which leads to electrostatic repulsion and disruption of the assembly. Both processes are reversible and importantly allow for the hierarchical structure of keratin (Figure 1a,b) to be restored at a later stage. Upon extraction with LiBr and DTT, a homogeneous solution is obtained, which we will refer to as “keratin homogeneous” (KH). Upon cooling this solution to 4 °C, KH spontaneously undergoes phase separation into two separate phases. Here, we will refer to the upper phase as “keratin supernatant” (KS), and the dense, bottom phase as “keratin bottom” KB (Figure 1d). This spontaneous phase separation is driven by the aggregation of different-sized peptides: keratins in KB have a larger molecular weight than the ones in KS, while both of them are present in KH.^19^ To quantify the protein concentration in the different phases, we dialyzed (3.5 kDa cutoff) them against water for 1 week, and freeze-dried them to obtain the pure keratin as a solid. The resulting protein concentrations in KH, KS, and KB were 1.6% (w/w), 1.1% (w/w), and 17.3% (w/w), respectively. This was confirmed using a Bradford protein assay. The total yield of keratin extraction was approximately 49% in weight.

### Coacervate Formation as a Function of pH

We explored the acidification of these protein solutions by adjusting their pH gradually from 7 to 1. We were surprised to observe that for pH values lower than 2.0, the solutions phase-separated, leading to the formation of a coacervate. Keratin is a protein with a strong tendency to self-interact and aggregate through different intermolecular forces, such as hydrophobic interactions and hydrogen bonds. Coacervate formation in keratin has already been observed upon complexation with a polyanion,^19^ and in the formation of KB itself. However, since in this case phase separation was clearly linked to a decrease in pH, we hypothesize that this process was related to the protonation of negatively charged groups in keratin, such as glutamate and aspartate residues,^33^ and the −COOH terminal ends of keratin (with p*K*_a_’s close to 2). We hypothesize that protonation of the carboxylic acid groups enables the formation of attractive interactions such as hydrogen bonds. Similar observations have also been reported for other biopolymers: recombinant spidroin shows increased attractive interactions and dimerization when the pH is lowered.^32^ Additionally, it has been observed that the viscosity of hyaluronan solutions reaches a maximum near pH 2.4, which has been ascribed to hydrogen-bond formation between carboxylic acid and acetamide groups.^33^ Additional factors may be at play, such as a lower affinity of the Li^+^ ions to the carboxylates upon protonation, leading to their detachment from the protein surface and reforming protein–protein associations.

The coacervates contained most of the keratin originally in solution, and their keratin content increased at a lower pH (Figure S1). Apparently, a lower pH leads to more effective coacervate formation and thus higher keratin extraction from the pristine keratin solution. These results were corroborated by the appearance of the coacervates (Figure 1e): all coacervates became more opaque upon lowering the pH. Phase separation was not observed above a pH of 2.0. Most likely this is due to the balance between the repulsive forces caused by absorbed Li ions and increased attraction forces induced by the pH drop.

### Structural and Material Properties of Coacervates as a Function of pH

To better understand the relationship between acidification and coacervation, we studied the thermal and structural properties of the formed keratin coacervates as a function of pH.

**Figure 2:**
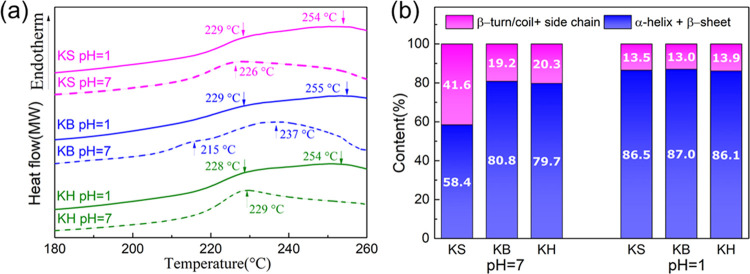
KS, KB, and KH before (pH = 7.0) and after (pH = 1.0) phase separation: (a) DSC curves and (b) analysis of the relative spectral contribution of secondary structures based on FTIR data.

The TGA profiles of KS, KB, and KH coacervates at pH 1.0 exhibited a higher thermostability than the corresponding solutions at pH 7.0 (shown in Figure S2). At pH 7.0, a major drop in weight, assigned to the denaturation of β-sheet crystallites and the degradation of the polypeptide backbone,^34^ was observed around 230 °C for all keratin coacervates (Figure S2). This weight loss was shifted to higher values of around 250 °C at pH 1.0 (after phase separation). We speculate that the improved thermostability of keratin in the coacervates may originate from the enhanced intermolecular interactions, especially hydrogen bonds and hydrophobic interactions.^35^ Additionally, in DSC experiments (shown in Figure 2a), a small shoulder was observed at around 228 °C in KS, KB, and KH coacervates for pH 1.0, corresponding to the denaturation of α-helix crystallites.^35^ We also noted a small peak in the DSC profile of KB at pH 7.0, assigned to a more ordered self-assembled structure due to the higher content in large peptides, as we have described previously.^19^

To probe the relative changes in the secondary structure of keratin molecules due to coacervation, we used Fourier transform infrared spectroscopy to study the amide(I) band (1600–1700 cm^–1^). The deconvolution of the amide(I) band is given in Figure S3, and the relative spectral intensities of the various components are summarized in Table S1. We first observe that the amide(I) bands of KS, KB, and KH coacervates (pH = 1.0 and pH = 1.7) all shifted to lower wave numbers after phase separation, suggesting the formation of intermolecular hydrogen bonds.^25^ The shoulder that appeared at 1625 cm^–1^ is attributed to intermolecular β-sheet formation. β-Sheets are very ordered structures, which typically increase the stability of proteins—explaining the improved thermostability observed in the TGA data. The contributions of ordered (α-helix + β-sheet) and disordered (β-turn/coil and side chain) secondary structures to the total spectral intensity are summarized in Figure 2b. These data suggest that at pH 1.0, KS, KB, and KH coacervates have a larger ordered fraction (giving rise to relative spectral intensities of 86.5, 87.0, and 86.1%, respectively) than the corresponding pristine keratin solutions before phase separation (where the corresponding relative spectral intensities amounted to 58.4, 80.8, and 79.7%) at pH 7.0. Particularly, upon phase separation, the combined relative spectral intensity of α-helices and β-sheets in the KS coacervate substantially increased from 58.4 to 86.5%, while that of the amorphous part decreased from 41.6 to 13.5%. This result agrees with other studies on spider silk and other biomolecular condensates, where a pH drop also facilitated conformational changes from amorphous to ordered domains.^25,26^

Additionally, changes in molecular interactions related to structural changes were also studied by X-ray photoelectron spectroscopy.^28^ As shown in Figure S4 and Table 1, deconvolution of nitrogen spectra revealed that the amount of hydrogen bonds for KB, KS, and KH keratin coacervates at pH = 1.0 slightly increased, compared to their counterparts at pH = 7.0. In agreement with the TGA and FTIR data, this indicates that a pH drop creates additional hydrogen-bond donors and promotes intermolecular interactions.^28^ We also found that KB has a higher content of hydrogen-bonded nitrogen than KS and KH, indicating a more ordered structure, in agreement with the FTIR analysis discussed above and previous results.^19^

**Table 1:** Relative Spectral Intensities of the Two Nitrogen Species, the NH Involved in Hydrogen-Bond Formation and the Bare NH in Polypeptide Backbone as Deduced from the XPS Spectra of the N 1s Core-Level Region of the Different Keratin Samples Prepared at pH = 1.0 and pH = 7.0

samples	–N–H···O=C– (401.8 eV) (%)	O=C–NH– (400.1 eV) (%)
KS, pH = 7.0	4.2	93.8
KS, pH = 1.0	5.4	94.6
KB, pH = 7.0	4.6	95.4
KB, pH = 1.0	7.2	92.3
KH, pH = 7.0	4.8	95.2
KH, pH = 1.0	5.5	94.6

**Figure 3:**
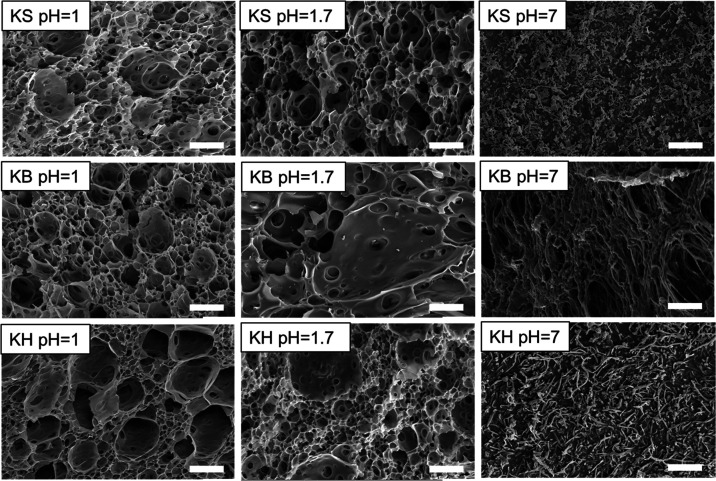
SEM images showing the morphology of keratin samples before (pH = 7.0) and after (pH = 1.0 and pH = 1.7) phase separation; the scale bar corresponds to 20 μm.

To investigate the influence of coacervation on the morphology of regenerated keratin, KS, KB, and KH prepared at different pH values were analyzed with SEM. As shown in Figure 3, interconnected porous morphologies were obtained for the pH 1.0 and 1.7 samples, which agrees well with other reported studies on complex coacervates, where porous structures appear upon desalting.^36^ However, for pH 7.0, a porous structure was only observed for the KB sample, not for KS and KH. We attribute this to the fact that KB is already phase-separated at pH 7.0, due to the low temperature in the extraction process.^18^

### Optimization of Spinning Dope

**Figure 4:**
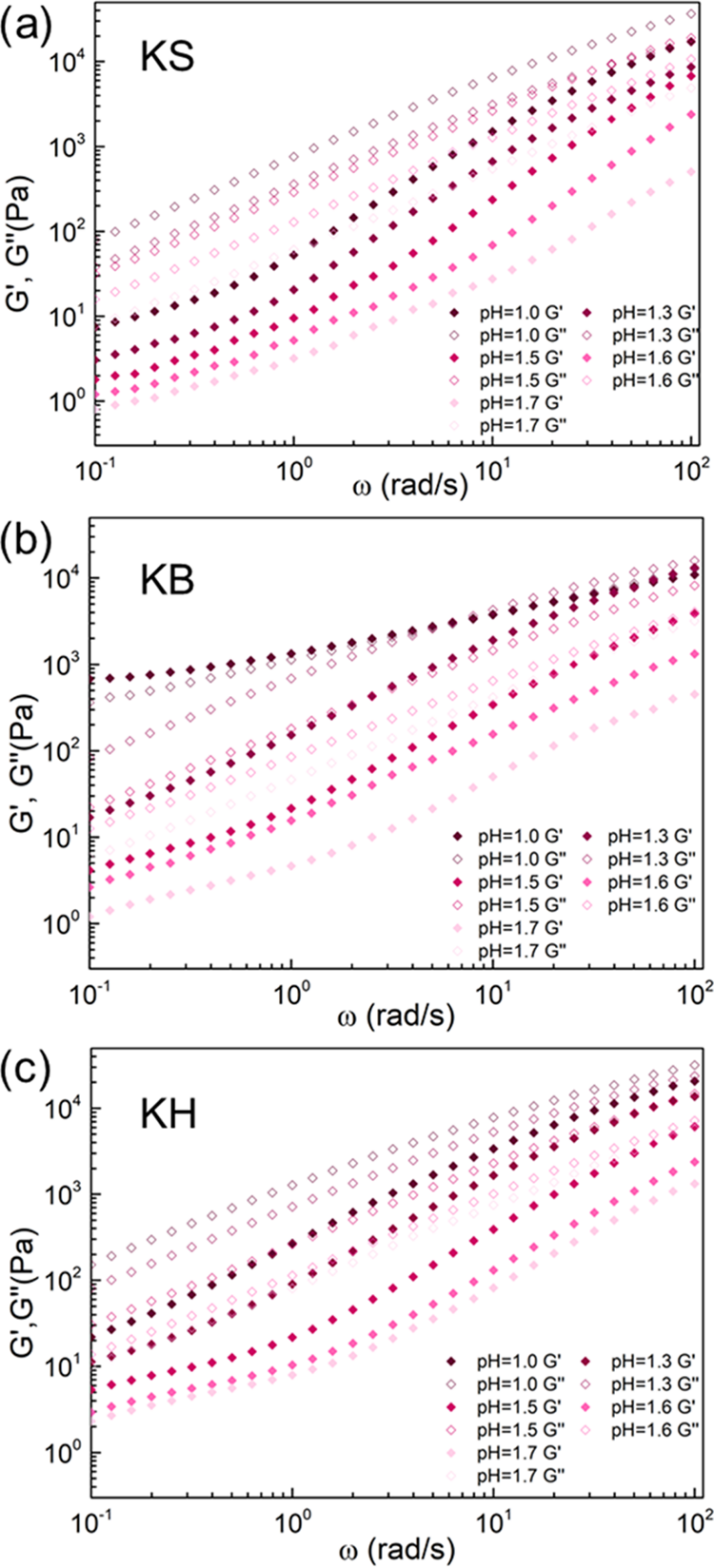
Viscoelastic properties of keratin coacervates as a function of pH: (a) KS; (b) KB; and (c) KH.

To optimize the spinnability of these coacervates, their viscoelastic properties were systematically investigated as a function of pH. The results of frequency sweep experiments, presented in Figure 4, show for all coacervates, i.e., KS, KB, and KH, typical features of a viscous liquid with the storage modulus (*G*′) lower than the loss modulus (*G*″) over almost the whole range of frequencies. This liquid-like viscous behavior is typical of coacervates and necessary for wet-spinning. Both *G*′ and *G*″ of KS, KB, and KH coacervates increase more than an order of magnitude when decreasing the pH from 1.7 to 1.0, probably due to the increased H-bonding between polypeptides at lower pH. Only for the KB coacervate at pH = 1.0, a crossover between *G*′ and *G*″ was observed at high frequencies, indicating a transition from liquid-like to solid-like behavior. These results demonstrate that the viscosity of keratin coacervates is tunable with pH, and therefore an optimal viscosity can easily be selected for each desired application. In our case, considering the viscoelastic properties and spinning apparatus that we used for wet-spinning, we decided that coacervates with a lower viscosity would be easier to extrude from the syringe pump while maintaining the stretchability of as-spun fibers. Therefore, we selected a pH of 1.7 for spinning.

To obtain solid fibers from these coacervates, we decided to make use of a deionized bath immediately after extrusion. In our design, when LiBr is diluted into the bath, it induces the solidification of the extruded material by a process commonly known as “salt-switch”.^37^ To test the feasibility of this approach, we performed a rheology experiment where we added deionized water around the coacervate and monitored the change in moduli during the resulting salt-switch. As shown in Figure S5, almost all keratin coacervates started out as a viscoelastic liquid (*G*″ > *G*′), yet quickly transitioned into a solid material (*G*′ > *G*″) upon contact with deionized water. The only exception was KB at pH 1.0 since it had solid-like properties before the salt-switch; however, also for this case, we observed a similar increase in *G*′ upon contact with deionized water. Since all samples had been diluted to the same concentration before pH switch, the rheological differences between KB and the other samples must be related to the higher molecular weight of KB.

The solidification process followed similar kinetics for all samples: first, they showed an abrupt increase in *G*′ (within the first 10 min in all cases), after which the moduli tended toward a plateau. These kinetics fit a process where the outer layer of the coacervate solidifies into a dense layer very quickly, which in turn slows down salt diffusion and leads to a coacervate with a porous inner structure. Morphologies of this type are typical for salt-switched coacervates^38−40^ and correspond to our experimental observations by SEM (Figure 3).

The crossover between *G*′ and *G*″ can be used as an estimate for the solidification (precipitation) time of the coacervate upon desalting. Interestingly, we observed that the precipitation time of the coacervate was inversely correlated to the pH in all cases: the coacervates that had a solid-like behavior before switching solidified much more slowly than the ones initially more liquid-like (Figure S6). We hypothesize that this effect is due to their differences in viscosity: low-pH coacervates are considerably more viscous, which slows down salt diffusion through them and therefore hinders their solidification.

### Mechanical Performance of Wet-Spun Keratin Fibers

**Figure 5:**
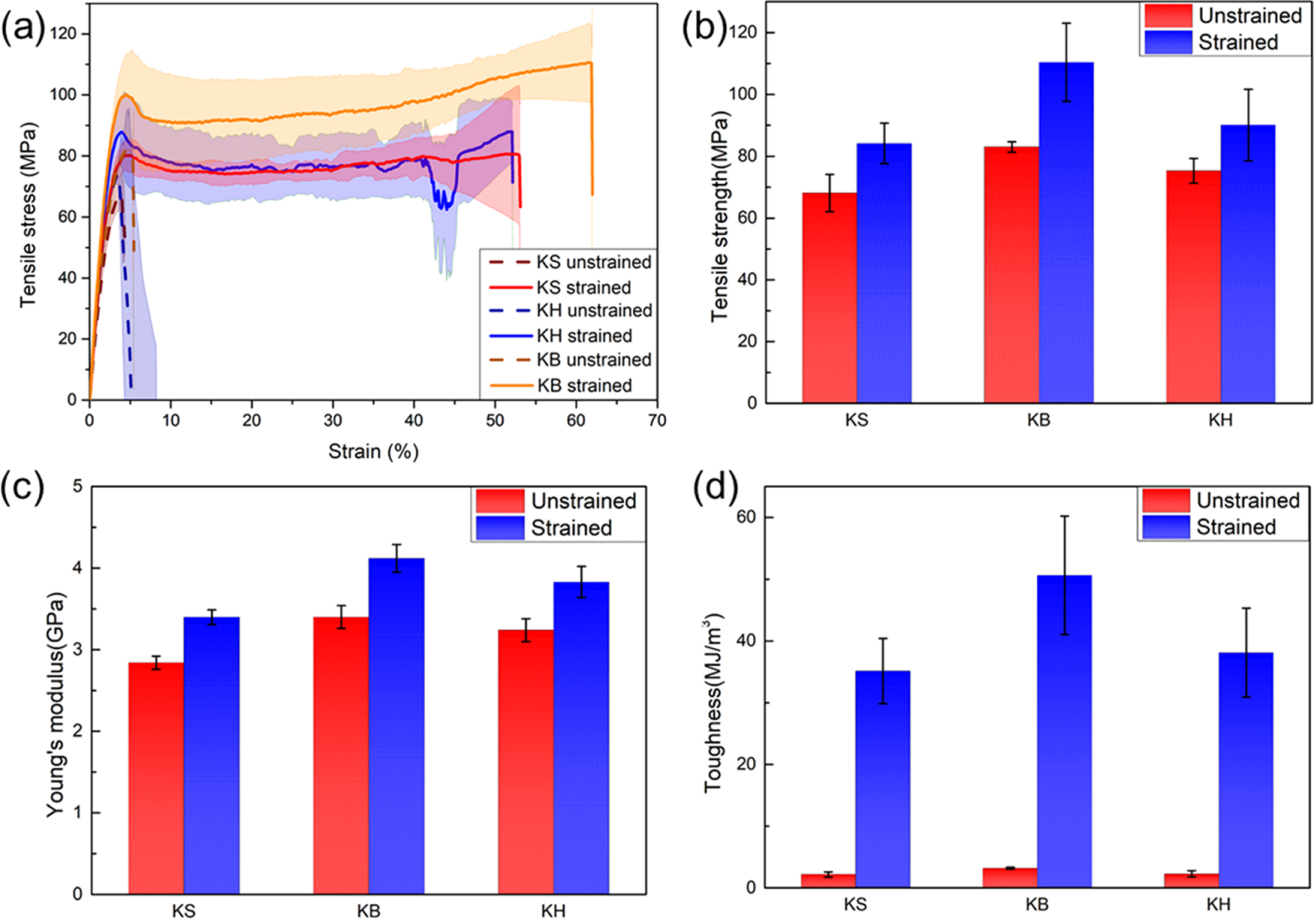
(a) Stress–strain curves of unstrained and strained keratin fibers. Mechanical performance of KS, KB, and KH: (b) tensile strength; (c) Young’s modulus; and (d) toughness. The shaded areas in (a) and the error bars in (b–d) correspond to the standard deviation of three samples.

As a proof of concept of the utility of these coacervates as intermediates for material formation, we prepared fibers through wet-spinning. As described in the previous section, we selected a pH of 1.7 and a coagulation bath based on deionized water. We added 1% H_2_O_2_ to the coagulation bath, to reform the disulfide bridges present in natural keratin, and further solidify the spun fibers (Figure 1c,f). To improve the mechanical properties of the fibers through alignment, a set of as-spun fibers were further stretched manually 1.5 times its original length (50%) and dried in air under tension afterward. The surface and cross section of fibers before and after drawing were studied with SEM. As shown in Figures S7 and S8, the porous structure characteristic of all coacervates (Figure 3) disappeared in the fibers, as a result of water evaporation. All stretched (KS, KB, and KH) fibers exhibited a significant increase in tensile strength, Young’s modulus, and in toughness (Figure 5a). Specifically, the ultimate strength and Young’s modulus were both increased by about 20% after drawing (Figure 5b,c), and the breaking strain was largely improved from 0.05 to 0.5.^41−44^ These two factors led to a substantial increase in toughness from ∼3 to 50 MJ m^–3^ (Figure 5d). We speculate that this improvement in mechanical properties may be attributed to changes in peptide conformation and/or orientation due to the drawing,^18^ which will be further investigated in the next section. The mechanical properties of strained keratin fibers were superior to the most previously reported values of keratin-based materials in the literature, as shown in Table S2.

The fibers from all keratin fractions had similar mechanical properties, with KB having slightly higher breaking stress, strain, and Young’s modulus. This can be explained by the higher content of high-molecular-weight proteins in KB, as determined previously,^19^ but the effect was minimal. These results show that for future applications, it is not necessary to induce phase separation of the high mass fraction of keratins (KB) after extraction—all of the extracted keratin can be directly used in the form of KH without impact on the final mechanical properties.

### Anisotropic Structure Enhanced by Drawing Treatment

**Figure 6:**
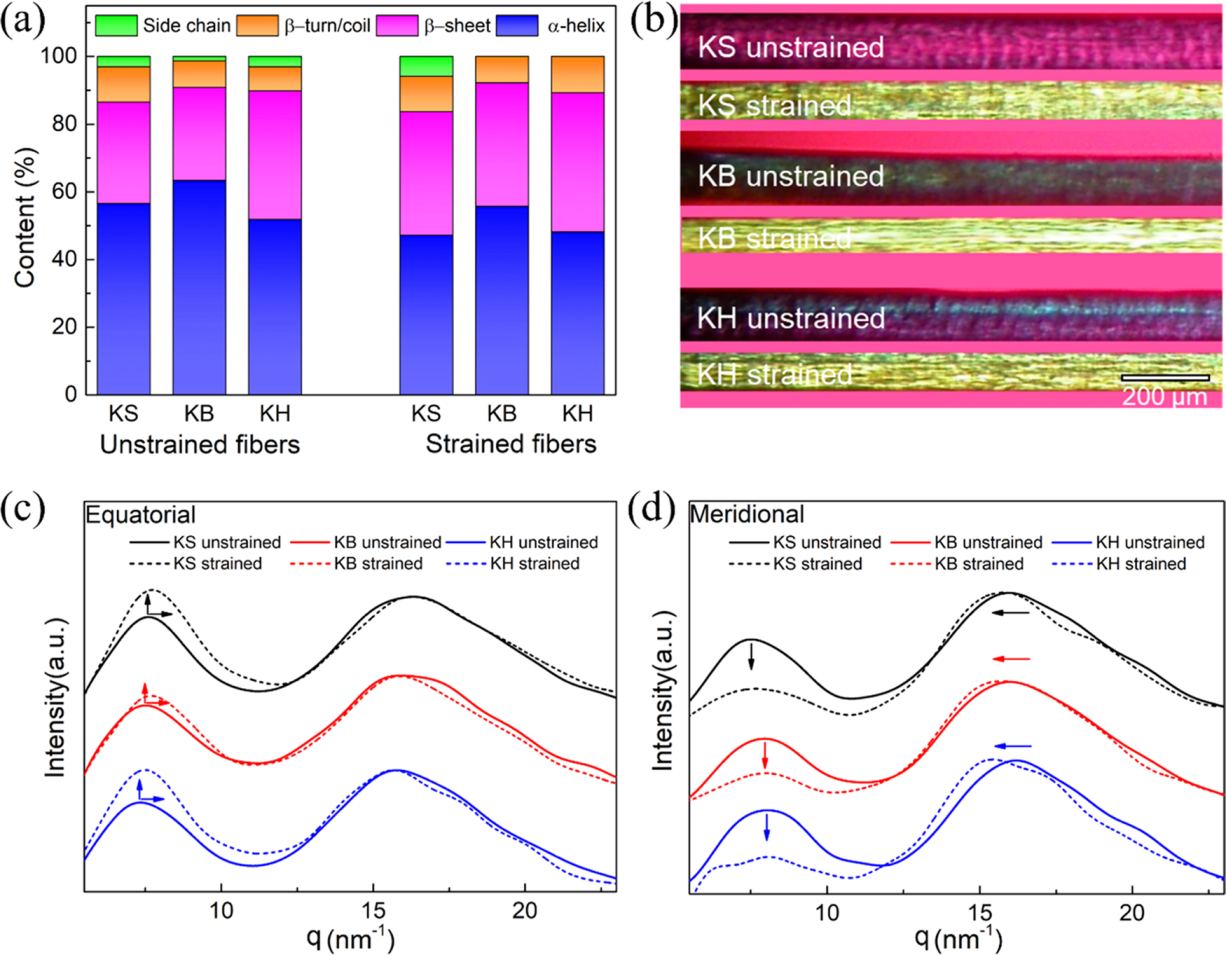
KS, KB, and KH keratin fibers before and after drawing: (a) analysis of the relative spectral contribution of the different secondary structures based on the FTIR data; (b) polarized light microscopy images showing the enhanced anisotropic birefringence after drawing. 1D scattering intensity profiles of radially integrated two-dimensional (2D) WAXS patterns both (c) in the equatorial direction and (d) in the meridional direction.

To better understand the effect of drawing on the structural properties of the regenerated keratin fibers, we characterized the secondary structure of keratin before and after drawing using FTIR. A possible drawing-induced transition from α-helix to β-sheet was monitored by tracking the shift of the amide(I) in the FTIR spectrum, which is known to have two characteristic bands, at 1620 cm^–1^ for β-sheets and 1650 cm^–1^ for α-helices. Spectral decomposition of amide(I) allowed us to derive qualitative conclusions and identify the trend of the relative spectral change of α-helix and β-sheet conformation in amide(I) band before and after drawing. The results for the peak deconvolution of the amide(I) band are shown in Figure S9 and summarized in Figure 6a and Table S3. After 50% drawing, a blueshift of the amide(I) was observed, and integration of the deconvoluted corresponding peaks demonstrated an increased contribution due to β-sheets and a decreased contribution due to α-helix strands to the spectral intensity for all strained KS, KB, and KH fibers. In earlier work, the conformational transition from α-helix to β-sheet was demonstrated to improve fiber mechanical properties, which can in part explain the improvement in the strength and toughness observed upon drawing.^18,45^

Polarized optical microscopy was used to provide insights into the structural orientation resulting from the drawing treatment. As shown in Figure 6b, the birefringence of strained KS, KB, and KH fibers was substantially increased after drawing, showing an increased anisotropic orientation of polypeptide chains along the fiber axis.

Wide-angle X-ray scattering analysis was used to obtain additional insights into the molecular conformation and anisotropic organization inside the fibers. The collected two-dimensional (2D) scattering patterns are isotropic for the unstrained (as-spun) keratin fibers, implying that no ordered structures with preferential alignment along the fiber axis are present (Figure S10). However, after 50% drawing, all strained keratin fibers showed an anisotropic pattern, confirming the presence of molecular orientation along the fiber axis. To quantitatively determine the evolvement of molecular conformation, the two-dimensional data for all keratin fibers were integrated in the direction perpendicular to the fibers (equatorial) and parallel to the fibers (meridional). As shown in Figure 6c,d, the characteristic equatorial reflections at *q* = 7.49 nm^–1^ and meridional reflections at *q* = 8.03 nm^–1^ correspond to the spacing between axes of adjacent α-helices in the two directions.^18,45^ The intensity corresponding to the spacing of α-helix strands greatly increased in the equatorial direction and decreased in the meridional direction upon drawing, indicating that the initially randomly distributed polypeptide chains were aligned parallel to the fiber axis upon drawing.^18^ The spacing between adjacent α-helices slightly decreased from 8.54 to 8.37 Å in the equatorial direction, pointing to a compaction of the helices, and the scattering peaks corresponding to the α-helix pitch slightly shifted to a lower *q* from 16.20 to 15.42 nm^–1^ in the meridional direction, indicating an increased spacing of the α-helix pitch from 3.88 to 4.07 Å. Taken together, the decreased spacing of the adjacent α-helix strands and the stretched α-helix pitch are both supportive of molecular alignment.

## Conclusions

Here, we have developed a green, benign, and extremely simple method to prepare regenerated keratin fibers from wool. Our protocol has three steps: extraction of keratin using aqueous solutions of LiBr, formation of a coacervate by lowering the pH, and wet-spinning into an aqueous bath containing H_2_O_2_ to reform the disulfide bridges from keratin. This protocol leads to materials with equal or superior properties to previously reported ones, while avoiding the traditional caveats related to the solution-based processing of keratin: multiple processing steps, elevated temperature, and organic solvents. It is worth mentioning that, although our method requires the use of high concentrations of LiBr, these salts are largely removed in the last step of the process. After wet spinning, the structure of the fibers is kept by intramolecular interactions and disulfide bonds, and the lithium and bromide ions are diluted into the aqueous bath, from which they can be potentially recovered.

An advantage of using pH-triggered coacervation over other methods is that it allows us to transform all of the extracted keratin into a spinning dope. Unlike other examples, this does not result in materials with significantly inferior mechanical properties, compared to the ones formed from only the largest peptides. In this work, we have focused on using the pH-induced coacervates as a spinning dope for fiber production, but we have also demonstrated that their mechanical properties can be tuned over a large range of values by changing the pH. Therefore, we envision that this method could easily fit other processing techniques where more liquid or solid “dopes” are required.

Finally, an additional advantage of the mild conditions used in this protocol is that the secondary structure of keratin is not denatured. As indicated by FTIR, WAXS, and SEM, the final fibers still retain a relatively ordered structure with a high content of α-helixes and β-sheets. This allows for the development of oriented hierarchical structures upon drawing, which result in mechanical properties that are comparable to synthetic fibers and natural silk. To the best of our knowledge, this combination of high conversion, simplicity, flexibility to tune the processing conditions, and potential to create materials with excellent mechanical properties is unprecedented for the processing of keratin. Therefore, we expect that this method can be used for the upcycling of waste keratin into high-value biocompatible products, and contribute to keratin recycling at a large scale.

## Supporting Information Available

Protein concentration of keratin coacervates varies with pH; TGA curves of keratin molecules before (pH = 7.0) and after (pH = 1.0) coacervation; FTIR spectra and peak deconvolution of amide(I) of keratin molecules at different pHs; summary of the relative spectral intensity content of secondary structures of keratin molecules at different pH; XPS spectrum of the N 1s core-level region of the three types of keratin; time sweeps of desalting process of keratin coacervates at different pHs; solidification time of keratin coacervates at different pH; SEM images of keratin fibers with and without stretching; SEM images showing the cross section of keratin fibers; comparison of mechanical properties of keratin-based materials; FTIR spectra and peak deconvolution of amide(I) of keratin fibers before and after stretching; summary of the relative spectral intensity of secondary structures of keratin molecules with and without drawing; and 2D WAXS scattering pattern obtained from unstrained and strained keratin fibers (PDF)
