# Supplementary material for: Bioinspired Processing of Keratin into Upcycled Fibers through pH-Induced Coacervation
Source: ACS Sustain Chem Eng. 2023 Jan 25;11(5):1985–94. doi: 10.1021/acssuschemeng.2c06865 (PMC9906721; doi:10.1021/acssuschemeng.2c06865)
Supplement: Supplementary file 1 — sc2c06865_si_001.pdf [file sc2c06865_si_001.pdf]

Bioinspired processing of keratin into upcycled fibers through pH-induced coacervation

Jianwu Sun [1], Guillermo Monreal Santiago [2], Feng Yan [3], Wen Zhou [4], Petra Rudolf [3], Giuseppe Portale [5], Marleen Kamperman [1]**

[1] Polymer Science, Zernike Institute for Advanced Materials, University of Groningen, Nijenborgh 4, 9747 AG, The Netherlands.

[2] Polymer Science, Zernike Institute for Advanced Materials, University of Groningen, Nijenborgh 4, 9747 AG, The Netherlands. Current affiliation: Université de Strasbourg, CNRS, UMR7140, 4 Rue Blaise Pascal, 67081 Strasbourg, France.

[3] Surfaces and Thin Films, Zernike Institute for Advanced Materials, University of Groningen, Nijenborgh 4, 9747 AG Groningen, The Netherlands.

[4] Products and Processes for Biotechnology, Engineering and Technology Institute Groningen, University of Groningen, Nijenborgh 4, 9747 AG, The Netherlands.

[5] Macromolecular Chemistry and New Polymeric Materials, Zernike Institute for Advanced Materials, University of Groningen, Nijenborgh 4, 9747 AG, The Netherlands.

Number of pages: 10

Number of figures: 10

Number of tables: 3

*Corresponding Authors:

Guillermo Monreal Santiago: monrealsantiago@unistra.fr

Marleen Kamperman: marleen.kamperman@rug.nl

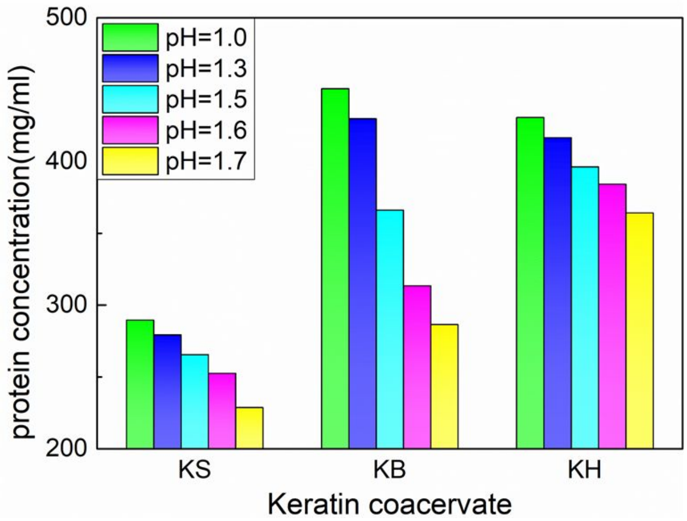

Figure S1. Protein concentration of keratin coacervates varies with pH. Here, the weight of keratin was obtained after dialysis and freeze-drying. The volume of coacervate was calculated by the deduction of the volume of supernatant from the total volume.

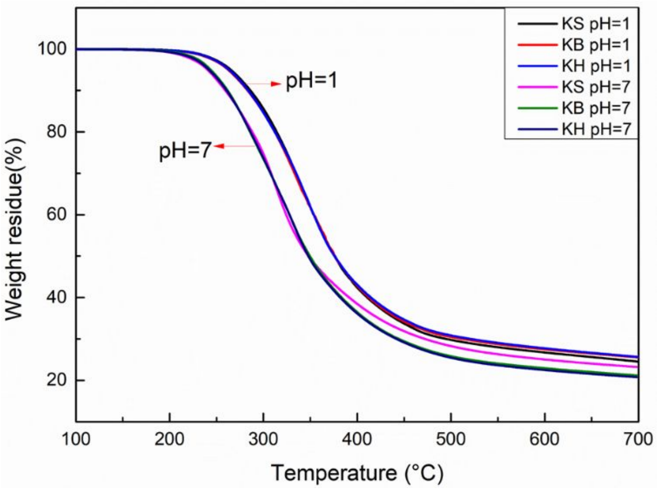

Figure S2. TGA curves of keratin molecules before (pH=7) and after (pH=1)

coacervation.

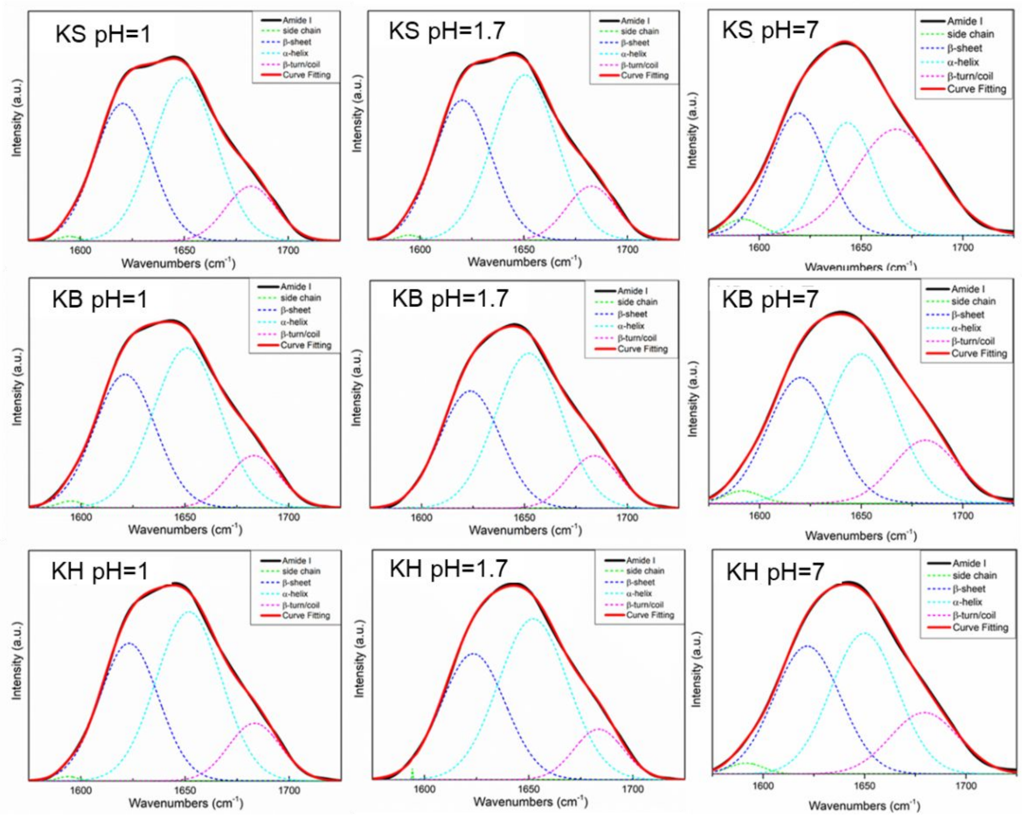

Figure S3. FTIR spectra and peak deconvolution of the amide I band region of keratin molecules prepared at different pH.

Table S1. Summary of the relative spectral intensity of secondary structures of keratin molecules prepared at different pH as deduced from the deconvolution of the amide I band region in the FTIR spectra.

		Side chain (%)	β-sheet (%)	φ-helix (%)	β turn/coil (%)
KS	pH=1.0	0.4	37.3	49.2	13.1
	pH=1.7	0.4	37.8	49.3	12.5
	pH=7.0	2.6	31.2	27.2	39.0
KB	pH=1.0	0.7	37.4	49.6	12.3
	pH=1.7	0.1	35.4	51.3	13.3
	pH=7.0	1.9	35.5	45.3	17.3
KH	pH=1.0	0.4	36.6	49.5	13.5
	pH=1.7	0	37.1	51.4	11.5
	pH=7.0	1.7	37.8	41.9	18.6

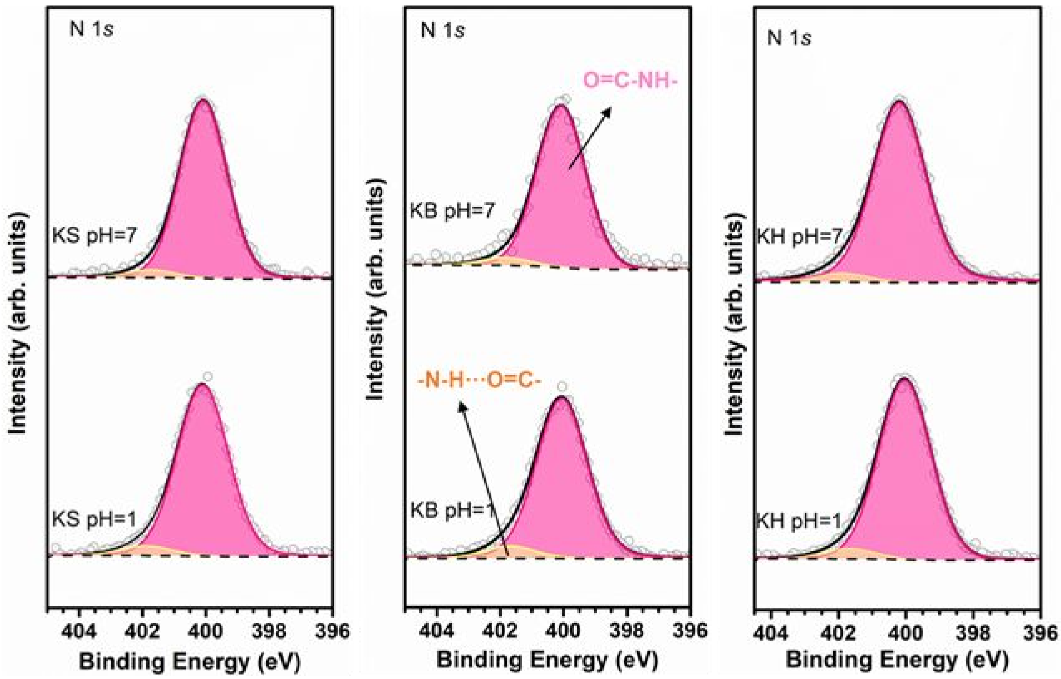

Figure S4. XPS spectrum of the N1*s* core level region of the three types of keratin, each prepared at different pH values, and corresponding fits. The peak deconvolution gives evidence for two different nitrogen species, NH involved in hydrogen-bond formation (-N-H···O=C-, at a binding energy of 401.8 eV) and NH in the polypeptide backbone (O=C-NH-, at a binding energy of 400.1 eV).

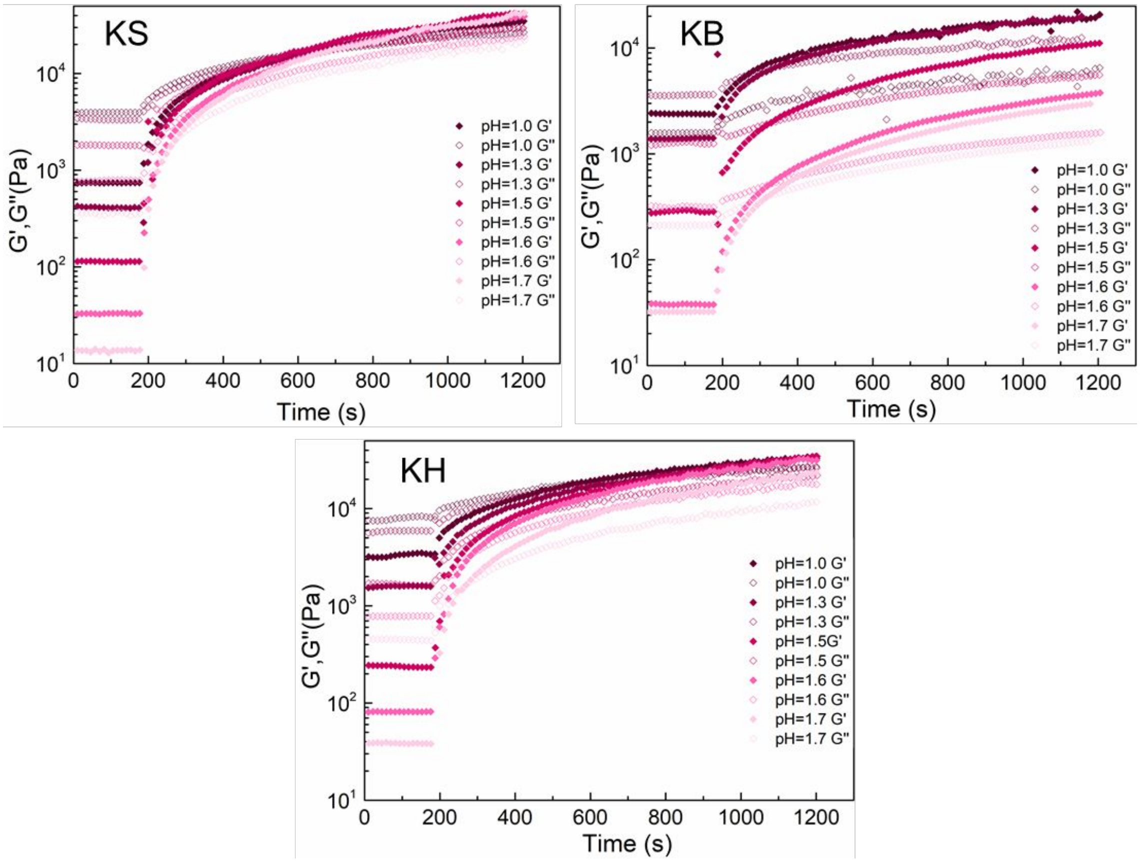

Figure S5. Time sweeps of the de-salting process of keratin coacervates at different pH.

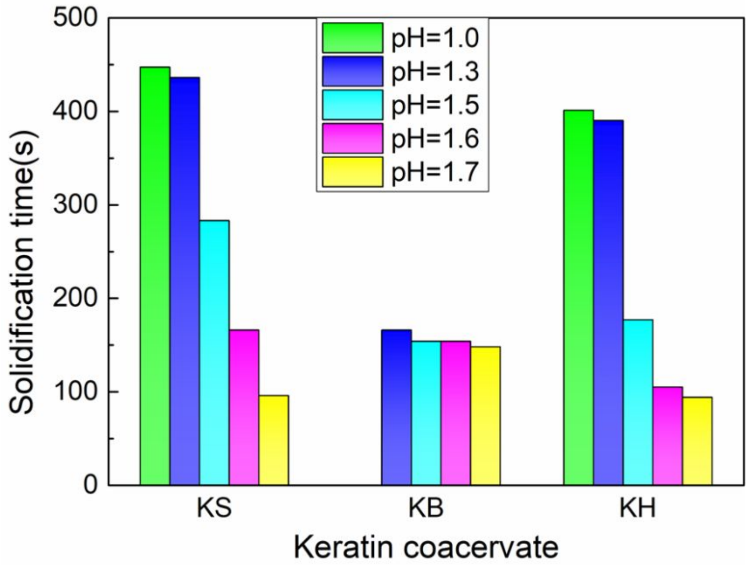

Figure S6. The solidification time of keratin coacervates at different pH.

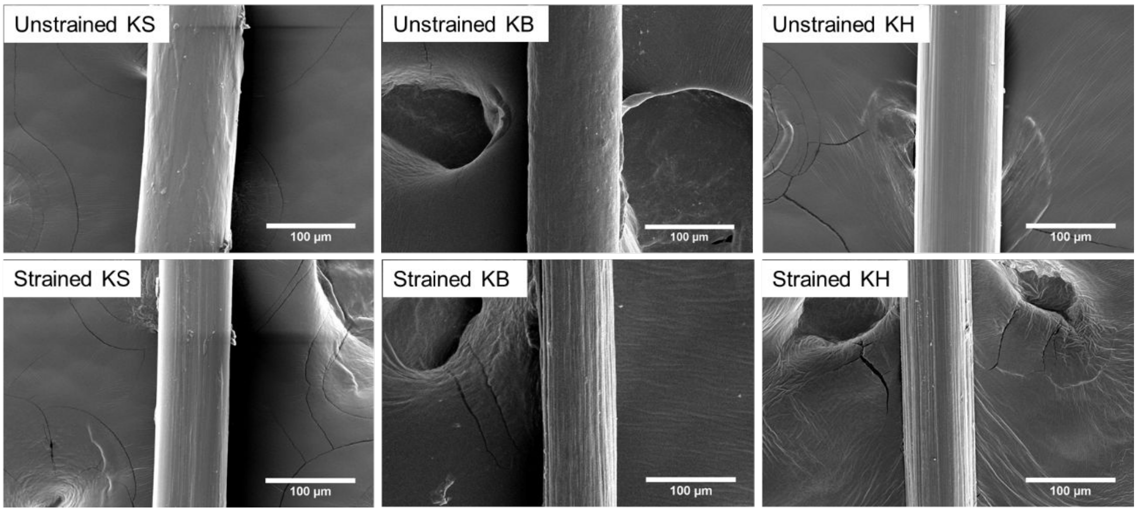

Figure S7. SEM images of keratin fibers prepared with (strained) and without (unstrained) stretching; the scale bar corresponds to 100 µm.

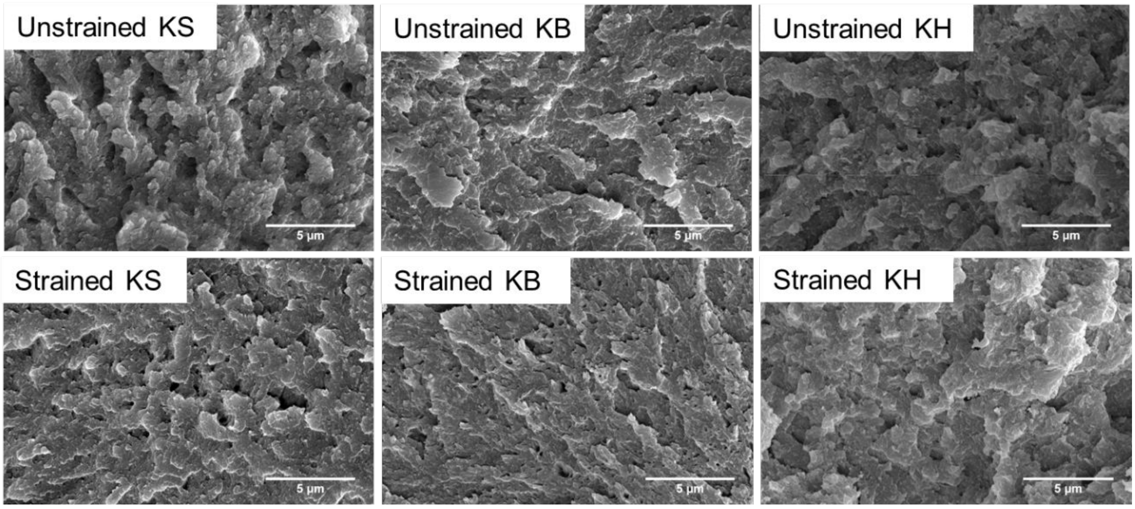

Figure S8. SEM images showing the cross-section of keratin fibers prepared with (strained) and without (unstrained) stretching; the scale bar corresponds to 5 µm.

Table S2. Comparison of mechanical properties of keratin-based materials between pH-induced coacervate keratin fibers obtained in this work and the previous reported values in the literature.

Materials	Keratin sources	Keratin (wt%)	Young's modulus (GPa)	Tensile strength (MPa)	Strain at break (%)
PVA/keratin fibers[1]	wool	25	/	5	41
PEO/keratin fibers[2]	wool	10-70	0.007-0.03	1.6-4.7	35-118
Cellulose/keratin fibers[3]	wool	80-95	2.8±0.5	29-87	12-80
Keratin fibers[4]	wool	100	/	101±15	11±3
Keratin fibers[5]	wool	100	4.2±0.1	>137	>85
This work	wool	100	4.1±0.2	110±12	63±5

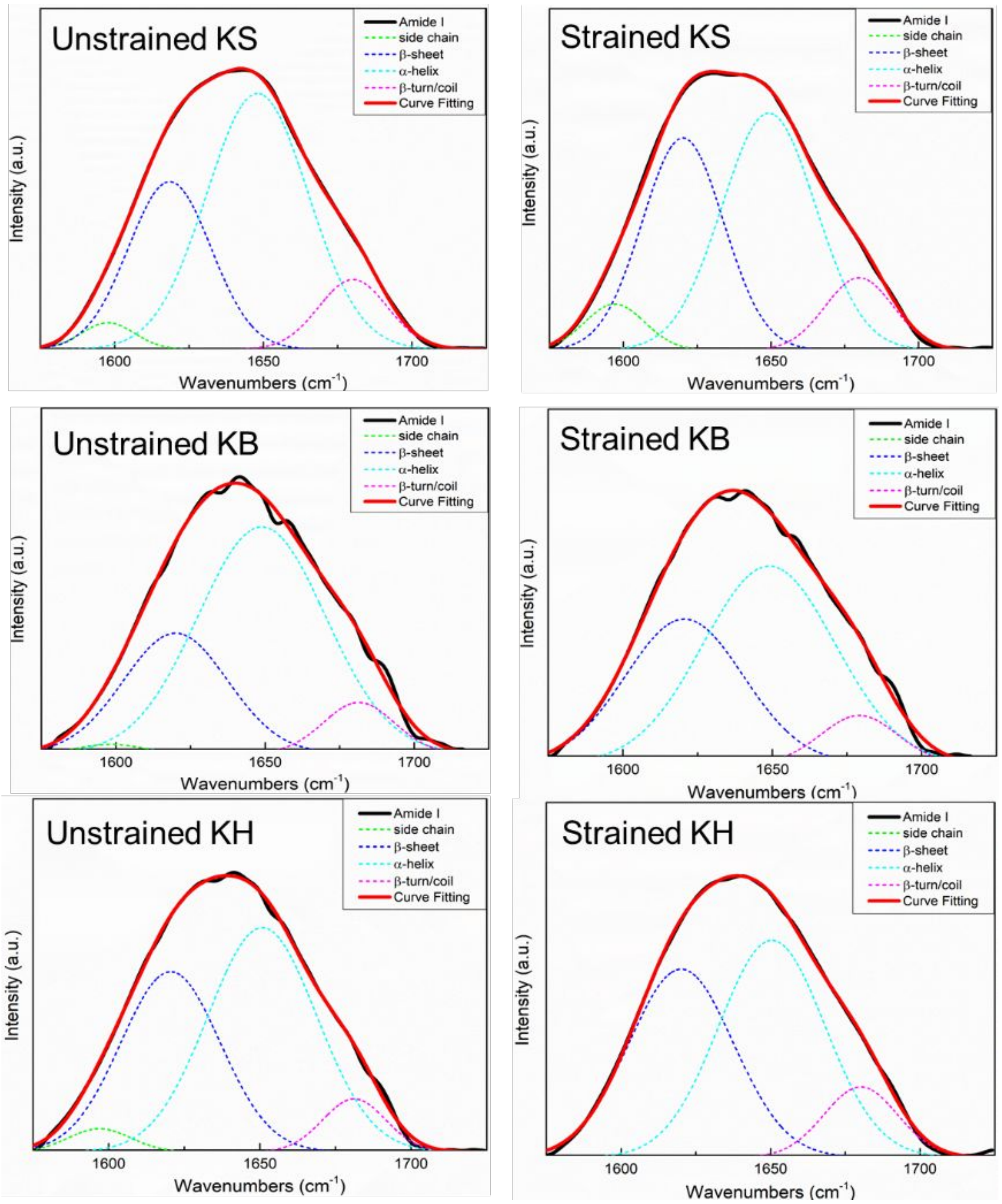

Figure S9. FTIR spectra and peak deconvolution of the amide I band region of keratin fibers before (unstrained) and after (strained) stretching.

Table S3. Summary of the relative spectral intensity of secondary structures of keratin molecules prepared with and without drawing, as deduced from the deconvolution of the amide I band region in the FTIR spectra.

		Side chain (%)	β-sheet (%)	α-helix (%)	β turn/coil (%)
KS	unstrained	3.1	30.0	56.5	10.4
	strained	5.9	36.5	47.2	10.4
KB	unstrained	1.3	27.5	63.3	7.9
	strained	0	36.5	55.7	7.8
KH	unstrained	3.1	38.0	51.8	7.1
	strained	0	41.1	48.1	10.8

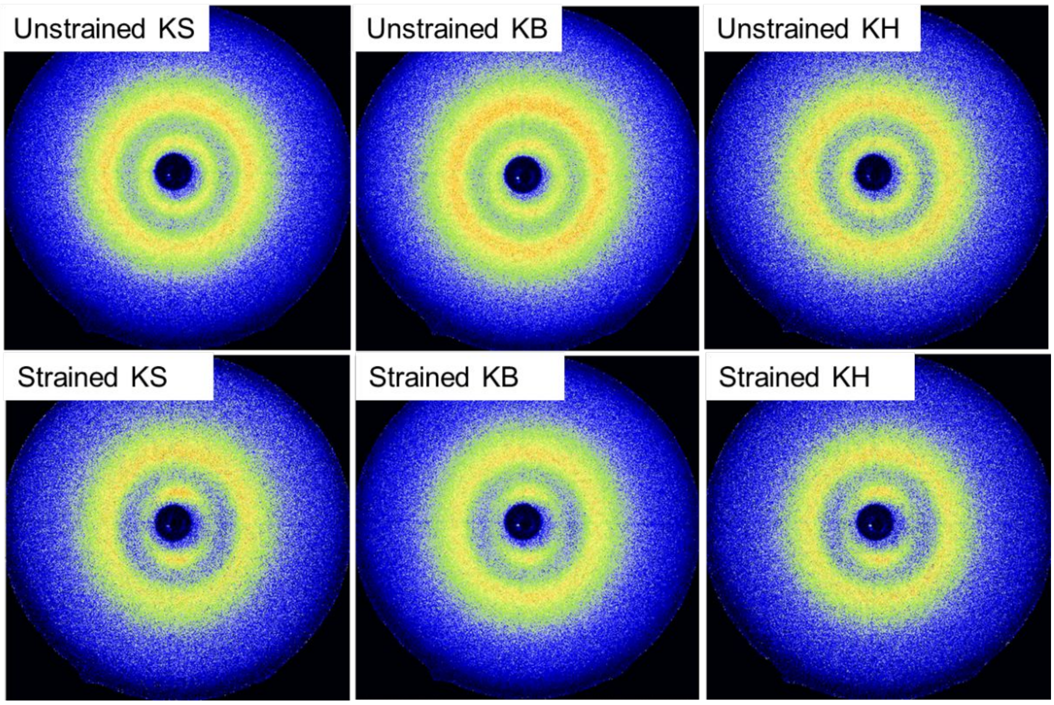

Figure S10. 2D WAXS scattering pattern obtained from unstrained and strained keratin fibers. The inner ring, related to the spacing between adjacent α-helix strands becomes anisotropic upon drawing.

Reference

1. Liu, R.; Li, L.; Liu, S.; Li, S.; Zhu, X.; Yi, M.; Liao, X., Structure and properties of wool keratin/poly (vinyl alcohol) blended fiber. *Adv. Polym Technol.* **2018,** *37* (8), 2756-2762.

2. Aluigi, A.; Vineis, C.; Varesano, A.; Mazzuchetti, G.; Ferrero, F.; Tonin, C., Structure and properties of keratin/PEO blend nanofibres. *Eur. Polym. J.* **2008,** *44* (8), 2465-2475.

3. Cao, G.; Rong, M. Z.; Zhang, M. Q., Continuous High-Content Keratin Fibers with Balanced Properties Derived from Wool Waste. *ACS Sustainable Chem. Eng.* **2020,** *8* (49), 18148-18156.

4. Xu, H.; Ma, Z.; Yang, Y., Dissolution and regeneration of wool via controlled disintegration and disentanglement of highly crosslinked keratin. *J. Mater. Sci.* **2014,** *49* (21), 7513-7521.

5. Cera, L.; Gonzalez, G. M.; Liu, Q.; Choi, S.; Chantre, C. O.; Lee, J.; Gabardi, R.; Choi, M. C.; Shin, K.; Parker, K. K., A bioinspired and hierarchically structured shape-memory material. *Nat. Mater.* **2021,** *20* (2), 242-249.
